# La_2_O_3_-CeO_2_-Supported Bimetallic Cu-Ni DRM Catalysts

**DOI:** 10.3390/ma16247701

**Published:** 2023-12-18

**Authors:** Pavel K. Putanenko, Natalia V. Dorofeeva, Tamara S. Kharlamova, Maria V. Grabchenko, Sergei A. Kulinich, Olga V. Vodyankina

**Affiliations:** 1 Department of Physical and Colloid Chemistry, National Research Tomsk State University, Tomsk 634050, Russia; putanenko.p@mail.ru (P.K.P.); nv-dorofeeva@yandex.ru (N.V.D.); kharlamova83@gmail.com (T.S.K.);; 2 Research Institute of Science and Technology, Tokai University, Hiratsuka 259-1292, Kanagawa, Japan

**Keywords:** bimetallic Cu-Ni catalysts, dry reforming of methane, complex LaCeO_x_ support, coking

## Abstract

The present work is focused on nickel catalysts supported on La_2_O_3_-CeO_2_ binary oxides without and with the addition of Cu to the active component for the dry reforming of methane (DRM). The catalysts are characterized using XRD, XRF, TPD-CO_2_, TPR-H_2_, and low-temperature N_2_ adsorption–desorption methods. This work shows the effect of different La:Ce ratios (1:1 and 9:1) and the Cu addition on the structural, acid base, and catalytic properties of Ni-containing systems. The binary LaCeO_x_ oxide at a ratio of La:Ce = 1:1 is characterized by the formation of a solid solution with a fluorite structure, which is preserved upon the introduction of mono- or bimetallic particles. At La:Ce = 9:1, La_2_O_3_ segregation from the solid solution structure is observed, and the La excess determines the nature of the precursor of the active component, i.e., lanthanum nickelate. The catalysts based on LaCeO_x_ (1:1) are prone to carbonization during 6 h spent on-stream with the formation of carbon nanotubes. The Cu addition facilitates the reduction of the Cu-Ni catalyst carbonization and increases the number of structural defects in the carbon deposition products. The lanthanum-enriched LaCeO_x_ (9:1) support prevents the accumulation of carbon deposition products on the surface of CuNi/La_2_O_3_-CeO_2_ 9:1, providing high DRM activity and an H_2_/CO ratio of 0.9.

## 1. Introduction

Currently, one of the urgent challenges in the chemical industry is the processing of greenhouse gases into valuable products. Processes based on CO_2_ and CH_4_ are of interest, since the content of these gases in the atmosphere increases annually and they are thus classified as renewable natural resources [1,2]. All of this allows us to consider them as raw materials for the process of dry reforming of methane (DRM), which is promising from the point of view of ecology- and resource-saving technologies. During this process, a synthesis gas (a mixture of CO and H_2_) is formed, which is widely used in industry to synthesize methanol, alcohols, components of synthetic hydrocarbon fuels, etc.:

CH_4_ + CO_2_ = 2CO + 2H_2_, (1)

DRM is a high-temperature process that occurs in the presence of catalysts based on noble metals (Pt, Pd, Ir, Rh, Ni, or Co). The most promising of these are the relatively cheap catalysts based on Ni, but they are characterized by a strong tendency to coking, which promotes their rapid deactivation [3,4,5,6,7,8]. Currently, the following strategies have been proposed to reduce such coking and increase the stability of Ni-based catalysts: (1) the use of supports, and (2) modifications with metals and oxides [3,4,5,6,7,9]. In Refs. [3,4,10,11], supports with a high concentration of oxygen vacancies or highly basic properties were proposed. In terms of these properties, the relatively accessible highly basic La_2_O_3_ [12] and CeO_2_ (which features high oxygen mobility [4,13,14]) are of interest, also demonstrating a strong metal–support interaction in the composition of nickel catalysts [4,15,16]. Despite the fact that both oxides are being actively studied as supports [3,5,14,16,17,18], there are few publications on La_2_O_3_-CeO_2_ binary systems. It is believed that the suppression of carbon deposition occurs through the formation of intermediate reactive products during the CO_2_ adsorption on the supports and their subsequent oxidation ability towards the conversion of highly active forms of carbon on the Ni particles. Dissociative adsorption occurs on CeO_2_ with the participation of surface oxygen vacancies (labeled as VOs) and Ce^3+^ cations [10,15,16,19,20,21,22,23,24,25]:

2Ce^3+^ + VO + CO_2_ = 2Ce^4+^ + O^2−^ + CO, (2)

Ni–C + O^2−^ + 2Ce^4+^ = Ni + VO + CO + 2Ce^3+^. (3)

Another mechanism of CO_2_ activation is characteristic of La_2_O_3_. Due to its high basicity, the lanthanum can bind CO_2_ on the surface, forming oxycarbonates [26,27]:

La_2_O_3_ + CO_2_ = La_2_O_2_CO_3_. (4)

Oxycarbonates exhibit high reactivity towards the oxidation of carbon deposition products [26,27,28,29]:

Ni–C + La_2_O_2_CO_3_ = Ni + La_2_O_3_ + 2CO. (5)

When studying these binary oxide systems, a synergistic effect can be assumed. In a wide range of La contents, the preservation of the crystal CeO_2_ structure is observed with an increase in the number of defects at high La contents [14,30] to imply that the mechanism with dissociative CO_2_ adsorption will predominate. At higher La contents, when La_2_O_3_ segregation is observed, one should expect the predominant formation of oxycarbonates, since the CeO_2_ addition enhances the basic properties of lanthanum [26,31,32] and the amount of Ce^3+^ sites required for dissociative adsorption is rather low. In both cases, a more effective CO_2_ activation and suppression of carbon deposition is expected than for the individual oxides, which makes these systems promising supports due to the opportunities to combine their properties that positively affect the stability and catalytic action of the Ni-containing catalysts. 

Transition metals (Fe, Co, Cr, Cu, Pt, Pr, Ir) or oxides of transition (MnO_2_, ZrO_2_, V_2_O_5_, CeO_2_, La_2_O_3_) and non-transition metals (CaO, MgO) are most often used as modifiers for Ni catalysts [3,4,5,6]. A promising direction is the use of small additions of transition metals, such as Fe [33], Co [34], or Cu [35,36,37]. These contribute to a greater dispersion of the active component, which is of fundamental importance for the resistance of Ni catalysts to coking [8]. The Cu modifier is considered the best option in terms of its price–quality ratio. A possible electronic effect was also indicated in [38]: since Cu itself did not contribute to the methane activation, the formation of the NiCu bimetallic species increased the activation barrier towards the dissociation of the C–H bond and, thus, inhibited the formation of the nuclei of the carbon phases. It is noteworthy that not just any Ni:Cu ratios result in lower carbon depositions [38,39]: at high Cu contents, a blocking of the surface sites of the activation of CH_4_ and mostly CO_2_ is observed, which leads to a decrease in the efficiency of the oxidation of the resulting carbon-containing particles and promotes a greater carbon deposition. The optimal Cu content has been found to be in the range of 10–30 mol.% [39], but it can be assumed that this range can be different when other methods of catalyst synthesis are used. There are few studies in the literature that combine the use of both supports and modifiers. The present work examines the influence of the composition of LaCeO_x_ supports (at La:Ce ratios of 9:1 and 1:1) and Cu additions on the catalytic properties of bimetallic Cu-Ni-supported catalysts and their stability to carbon deposition in the DRM process. The La:Ce ratios in this study were chosen according to different pathways of CO_2_ activation by the surface of the support enriched with a La_2_O_3_ or LaCeO_x_ solid solution.

## 2. Materials and Methods

### 2.1. Preparation of Supports and Catalysts

The citrate method was used to prepare the supports. La(NO_3_)_3_∙6H_2_O (chemically pure, “Rare Metals Plant”, Koltsovo, Russia), Ce(NO_3_)_3_∙6H_2_O (analytical grade, “Soyuzkhimprom”, Novosibirsk, Russia), and C_6_H_8_O_7_∙H_2_O (chemical grade, “Khimprom”, Moskow, Russia) were used as precursors. Citric acid monohydrate was taken in such a quantity that the total number of moles of citric acid was 1.2 times greater than the total number of moles of La and Ce. The solution was prepared by mixing the concentrated solutions of precursors, and was kept under continuous stirring and heating (75 °C) for 3 h. The resulting viscous mass was dried stepwise from 80 to 120 °C, then calcined at 700 °C for 4 h. In this way, two supports were prepared with La:Ce ratios of 1:1 and 9:1 (hereinafter referred to as LaCeO_x_ 1:1 and LaCeO_x_ 9:1). Pure La_2_O_3_ and CeO_2_ were also synthesized as reference samples.

The active components were supported by wet impregnation based on moisture capacity with the solutions of Ni(NO_3_)_2_∙6H_2_O (analytical grade, “Reakhim”, Moskow, Russia) and Cu(NO_3_)_2_∙3H_2_O (analytical grade, “Reakhim”, Moskow, Russia). The nominal Ni and Cu contents were 10 wt.% and 2.2 wt.%, respectively, which corresponded to the molar ratio Ni:Cu = 1:0.2. The soaked samples were dried at room temperature and calcined for 4 h at a temperature of 700 °C.

The synthesized catalyst samples were designated as NiCu(X)/LaCeO_x_ Y:Z (where X is the mole fraction of Cu relative to Ni, and Y:Z is the molar ratio of La to Ce, respectively): NiCu(0)/LaCeO_x_ 1:1, NiCu(20)/LaCeO_x_ 1:1, NiCu(0)/LaCeO_x_ 9:1, NiCu(20)/LaCeO_x_ 9:1.

### 2.2. Characterization Methods

The elemental compositions of the supports and catalysts were determined using the XRF-1800 sequential wave-dispersive X-ray fluorescence spectrometer (Shimadzu, Kyoto, Japan).

The phase composition of the samples was studied via X-ray phase analysis (XRD) using a Shimadzu-7000 diffractometer (Shimadzu, Kyoto, Japan) with a Cu anode (λ = 1.5418 Å) in the 2θ range from 10 to 80°, with a scanning rate of 20 deg/min. Identification of the phase composition was carried out using the PCPDFWIN and ICSD databases. The dimensions of the coherent scattering regions (CSRs) were determined using the Scherrer equation:

(6) $$d=\frac{K\lambda }{\beta \cos\theta },$$

where d is the crystallite diameter, nm; K is the factor (0.94); λ is the wavelength, nm; β is the reflection width at half height; and θ is the diffraction angle, rad.

The textural properties of the samples were studied using the method of low-temperature nitrogen adsorption–desorption. These studies were carried out using the Tristar 3020 specific surface area analyzer (Micromeritics, Norcross, GA, USA). Measurements were carried out after the preliminary degassing of the samples under vacuum conditions at a temperature of 200 °C for 2 h. The SSA was determined using the multipoint BET method. The porosity of the samples and the nature of the pore size distribution were determined by analyzing the desorption branch of the isotherms using the BJH desorption method.

To evaluate the acid base properties of the surface of supports and catalysts, the method of temperature-programmed CO_2_ desorption (TPD-CO_2_) was used. These studies were carried out using the automatic chemisorption analyzer AutoChem HP 2950 V2.03 (Micromeritics, Norcross, GA, USA). The samples were preheated in a stream of air (for the supports) or a mixture of 10 vol.% H_2_/Ar (for the catalysts) up to 700 °C, followed by cooling to 100 °C. The CO_2_ adsorption was carried out in isothermal mode at a temperature of 100 °C for 30 min using a mixture of 5 vol.% CO_2_/He. Then, the reactor was purged with He and the desorption curve was recorded with a linear increase in temperature at a rate of 10 °C/min in the range of 50 to 900 °C. Registration of the released gases was carried out using a thermal conductivity detector (TCD) and the UGA300 quadrupole mass spectrometer (Stanford Research Systems, Lake Mary, FL, USA).

To evaluate the temperature ranges for the reduction of the surface components of the catalysts, the method of temperature-programmed reduction with hydrogen (TPR-H_2_) was used. The analysis was carried out on the AutoChem HP 2950 V2.03 chemisorption analyzer (Micromeritics, Norcross, GA, USA). Pretreatment of the samples was carried out in a flow of air when heated to up to 700 °C. The recovery curve was recorded during the linear heating of the samples in a mixture of 10 vol.% H_2_/Ar up to 850 °C at a rate of 10 °C/min. Hydrogen consumption was recorded using the TCD.

The catalytic properties of the samples were studied in a quartz flow reactor with a stationary catalyst bed with an online analysis of the reaction mixture. A total of 50 mg of catalyst (0.125–0.25 mm fraction) diluted with quartz chips (1 cm^3^, 0.5–1.0 mm fraction) was placed into the reactor. Prior to the analysis, the sample was reduced in a flow of 50 mL/min of a 10 vol.% H_2_/N_2_ at 700 °C for 1 h, followed by cooling down to 400 °C. A gas mixture of 14 vol.% CH_4_ and 14 vol.% CO_2_ in N_2_ was used as the reaction mixture.

The temperature’s effect on the catalytic properties of the obtained samples was studied in the range of 400 to 800 °C (in steps of 100 °C or 50 °C; the holding time was 30 min at each target temperature). The stability of the catalysts was studied in a series of isothermal tests at 650 °C for 6 h. The reaction mixture was analyzed using the Kristall 5000.2 chromatograph (“Khromatek”, Yoshkar-Ola, Russia) with a flame ionization detector in combination with a methanator and a TCD. The mixture components were separated using the NaX packed column to determine H_2_ and N_2_, and the PoraPlot Q capillary column to determine CO, CO_2_, and CH_4_. 

Conversions of CH_4_ and CO_2_ and carbon balance (B) were calculated according to the following formulas:

(7) $$X\left(CH_{4}\right)=\frac{C_{{\mathrm{CH}}_{4}\;}^{0}-(C_{{\mathrm{CH}}_{4}}\cdot C_{N_{2}}^{0}/C_{N_{2}})}{C_{{\mathrm{CH}}_{4}}^{0}}\cdot 100\%\;,$$

(8) $$X\left(CO_{2}\right)=\frac{C_{{\mathrm{CO}}_{2}}^{0}-(C_{{\mathrm{CO}}_{2}}\cdot C_{N_{2}}^{0}/C_{N_{2}})}{C_{{\mathrm{CO}}_{2}}^{0}}\cdot 100\%,$$

(9) $$B=\frac{C_{{\mathrm{CO}}_{2}}+C_{{\mathrm{CH}}_{4}}+{\;C}_{\mathrm{CO}}}{{C_{{\mathrm{CO}}_{2}}^{0}+C}_{{\mathrm{CH}}_{4}}^{0}}\cdot \frac{\nu_{\mathrm{out}}}{\nu_{\mathrm{in}}}\cdot 100\%$$

where C_i_ is the concentration of the i^th^ component at the reactor outlet, mol/L; C_0i_ is the concentration of the i^th^ component at the reactor inlet, mol/L; υ_in_ and υ_out_ are the volumetric flow rates before and after the reactor, mL/min. The i^th^ components include CH_4_, CO_2_, CO, and N_2_.

Qualitative and quantitative analyses of the carbon deposition products were carried out after the stability tests. The qualitative analysis of the structure of carbon deposition products was carried out on the In-Via confocal Raman dispersive spectrometer (Renishaw, New Mills, UK) equipped with a Leica microscope with a 50× objective. Excitation was carried out with a solid-state Nd:YAG laser with a wavelength of 532 nm and a radiation power of 100 mW. Raman spectra were measured in the range of 100–2000 cm^−1^ with a spectral resolution of 2 cm^−1^. The amount of carbon deposition products was determined using a synchronous thermal analyzer (STA): STA 449 F1 Jupiter (NETZSCH, Selb, Germany). The measurements were carried out in an atmosphere of a N_2_/O_2_/Ar mixture and a temperature ranging from 25 to 900 °C, with a heating rate of 10.0 °C/min.

## 3. Results

### 3.1. Composition and Structure of Catalyst Precursors

Table 1 shows the results of our quantitative analysis, the determination of textural properties and phase composition of the supports and catalyst precursors.

According to the XRF data, the La:Ce ratios match the nominal values. The Ni contents are close to the nominal ones as well, while the Cu contents slightly increase. The supports and catalysts are characterized by small specific surface area and pore volume; the average pore diameter indicates the mesoporous texture of the samples. Similar textural features are characteristic of oxide ceramics after high-temperature treatment of gels [40,41]. The formation of the porous structure of the supports occurs as a result of the release of gases through the gel structure.

**Figure 1:**
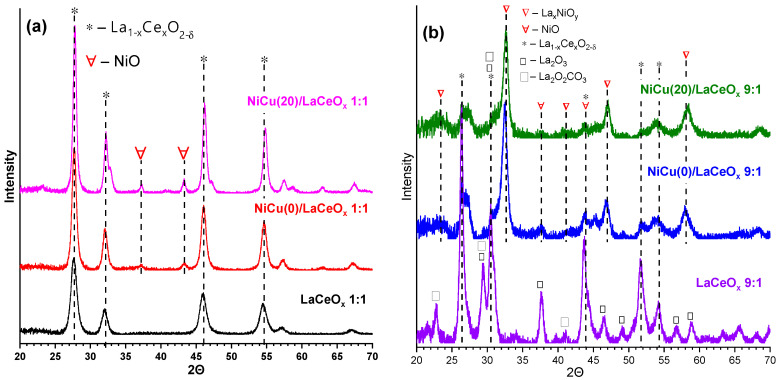
XRD patterns for supports and precursors of Cu-Ni-containing catalysts prepared on the basis of: (**a**) LaCeO_x_ 1:1; (**b**) LaCeO_x_ 9:1.

Figure 1 shows the XRD patterns for the obtained supports and catalyst precursors.

For the support with the La:Ce = 1:1 ratio (see Figure 1a), we observe a preservation of the relative intensity and relative position of the reflections characterizing the CeO_2_ phase, which indicates the formation of the LaCeO_x_ substitution solid solution with the preservation of the crystal structure of CeO_2_. The shift in the reflections towards the region of lower 2Θ indicates an increase in the unit cell parameter, from 5.41 (for pure CeO_2_) to 5.58 Å.

For the support with the La:Ce = 9:1 ratio (see Figure 1b), additional reflections appear, indicating the formation of new phases. These reflections are related to the phases of individual La_2_O_3_ oxide with hexagonal symmetry and the carbonate La_2_CO_5_ formed by binding CO_2_ released during the combustion of citric acid. This is consistent with data from the literature on the segregation of lanthanum oxide on the surface of LaCeO_x_ solid solutions at high La:Ce ratios (≥0.8) [26]. For this support, an increase in the unit cell parameter to 5.85 Å is observed, which apparently is the limiting value above which the La_2_O_3_ segregation occurs.

The calculated CSR sizes (see Table 1) are 10 nm and 22 nm for the LaCeO_x_ solid solution of the 1:1 and 9:1 series, respectively. The dimensions of the La_2_O_3_ CSR segregated in the 9:1 sample are 17 nm. These obtained values are consistent with their textural properties: the LaCeO_x_ 9:1 support that demonstrates large CSR sizes has smaller specific surface area values relative to the LaCeO_x_ 1:1. This allows us to assume that the dimensions of the CSR are formed at the stage of gas evolution, and during the subsequent sintering upon calcination they change only slightly, although this issue has not been reported in detail in the literature to date.

For the catalyst precursors of the 1:1 series (see Figure 1a), the preservation of all reflections related to the phase of the LaCeO_x_ solid solution is observed. Reflections related to the NiO phase also appear. In Ref. [26], a formation of the Ce_1-x-y_La_x_Ni_y_O_2-δ_ solid solution is reported for similar systems by decreasing the unit cell parameter of the LaCeO_x_ (shifting the reflections towards higher 2Θ angles). Our results confirm its presence and indicate strong evidence of the precursor–support interaction. The reflections of Cu-containing phases are not detected in all Cu-containing samples, which indicates either a high dispersion of the CuO phase or the formation of a NiCuO_x_ solid solution that retains the NiO structure [42]. No shift in the NiO-related reflections is observed in the studied samples, which can be due to the small amounts of components.

The application of the active component is accompanied by an increase in the CSR of the solid solution to up to 12–15 nm (see Table 1), which can be explained by the sintering of the particles during the secondary calcination. The Cu addition affects both the dispersions of the solid solution and NiO.

The phase composition of the catalyst precursors of the 9:1 series is more complicated than that of the 1:1 series (see Figure 1b). The structure of the LaCeO_x_ solid solution is preserved and it is possible to identify the reflections for the NiO phase; however, the reflections of a complex mixture of perovskite lanthanum nickelates (containing compounds such as LaNiO_3_, La_2_NiO_4_) appear when the Ni-containing precursors interact with the segregated La_2_O_3_ on the surface of crystallites of the solid solution [26,43,44].

Based on the ratio of the intensities of the LaNiO_x_ and LaCeO_x_ reflections, the lanthanum nickelates are the main phases in the unreduced catalyst in terms of the mass content. Since there is less segregated La_2_O_3_ in the initial support than in the solid solution, it can be assumed that the additional segregation of La_2_O_3_ from the subsurface layers of the support occurs as a result of its strong interaction with the Ni precursor.

No copper-containing phases were found in the composition of the unreduced catalysts; however, according to the data on one-pot systems [18,45,46], copper is also prone to forming perovskite-like oxide compounds with lanthanum. Detection of these phases is difficult due to the probable formation of nickelate-cuprates or the overlapping of reflections of the individual compounds.

### 3.2. Formation of Active Component, Structure, and Acid Base Properties of the Reduced Catalysts 

**Figure 2:**
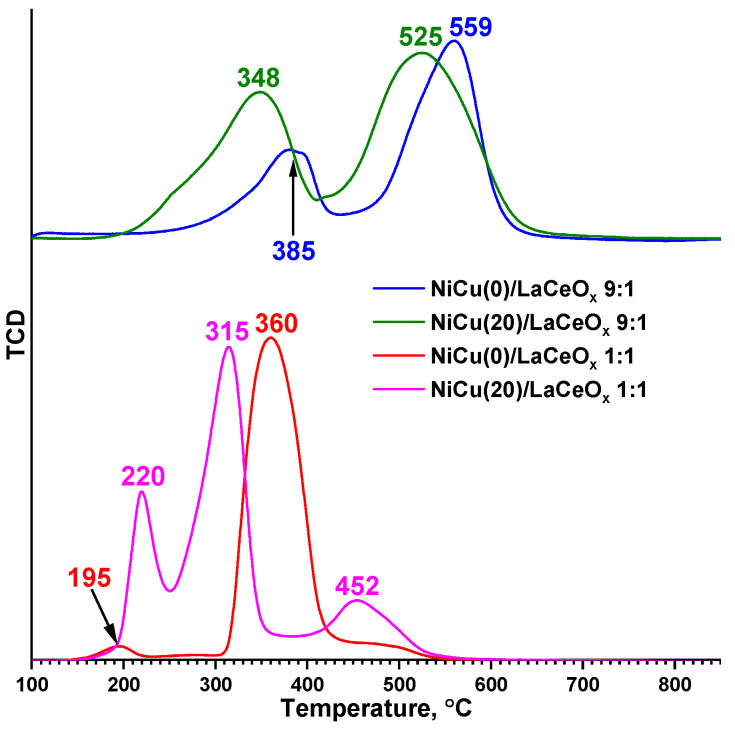
TPR-H_2_ profiles for catalysts.

Figure 2 and Table 2 show the TPR-H_2_ data characterizing the process of reduction for the precursors and the formation of the active component.

For the 1:1 series, the presence of a peak with a maximum temperature in the region of 315–360 °C is observed, which is related to the reduction of NiO and the Ce_1-x-y_La_x_Ni_y_O_2-δ_ solid solution [26,36,47]:

NiO + H_2_ = Ni^0^ + H_2_O, (10)

Ce_1-x-y_La_x_Ni_y_O_2-δ_ + yH_2_ = Ce_1-x-y_La_x_O_2-δ-y_ + yNi^0^ + yH_2_O. (11)

A peak with a low temperature maximum at 195 °C can be attributed to the reduction of oxygen species adsorbed on the vacancies associated with the Ce_1-x-y_La_x_Ni_y_O_2-δ_ solid solution [48].

The peak of hydrogen consumption at 220 °C is associated with the reduction in Cu-containing species in the NiCu(20)/LaCeO_x_ 1:1 sample, as is observed in Ref. [47]:

CuO + H_2_ = Cu^0^ + H_2_O. (12)

The appearance of a peak at 452 °C indicates the presence of highly dispersed nickel oxide particles that strongly interact with the support [16].

The 1:1 series is characterized by a decrease in the NiO reduction temperature as the Cu content increases. The proposed mechanism of its action is as follows: copper is the first to be reduced when heated, according to reaction 12. The resulting Cu^0^ species become the sites for dissociative adsorption of hydrogen, and the hydrogen spillover onto NiO particles occurs as a result in the surface reduction of the oxide [47]. Thus, copper decreases the reduction temperature of nickel oxide with the more efficient adsorption and activation of H_2_ in comparison with the catalyst without Cu addition. For such a mechanism, the Cu atoms should be sufficiently close to those of nickel, which is possible in two cases: where Cu is on the surface either in the form of ultra-dispersed CuO oxide particles, or in the form of a NiCuO_x_ solid solution. The latter case is supported by the formation of bimetallic NiCu particles [38,47,49] with the Cu segregation on their surface [38].

For the 9:1 series, two temperature maxima of hydrogen consumption are observed, which are attributed to the stepwise reduction of nickelates stabilized in the oxidation states +2 and +3 [44,50]:

2LaNi**^III^**O_3_ + H_2_ = La_2_Ni^II^_2_O_5_ + H_2_O, (13)

La_2_Ni^II^_2_O_5_ + H_2_ = 2Ni^0^ + La_2_O_3_ + 2H_2_O, (14)

La_2_Ni^II^O_4_ + H_2_ = Ni^0^ + La_2_O_3_ + H_2_O. (15)

This scheme is conditional since the exact composition of nickelates on the surface of the catalysts is rather difficult to identify. Reaction 13 occurs in the region of 350–400 °C, while reactions 14 and 15 proceed in the high-temperature region. It is also likely that the small amounts of detected NiO are reduced according to reaction 10. As a result of these reactions, the perovskite structures of nickelates are completely destroyed, and Ni^0^ and La_2_O_3_ are released, which is consistent with the XRD data (see Figure 1b). Similar schemes were proposed for the reduction of lanthanum cuprates in [46,51]:

LaCu^II^O_3_ + H_2_ = LaCuO_2_ + H_2_O, (16)

LaCu^I^O_2_ + H_2_ = Cu^0^ + La_2_O_3_ + H_2_O. (17)

Reactions 16 and 17 occur in the regions of 306 °C and 500 °C, respectively. Since Cu is formed only at high temperatures, it can be assumed that the spillover effect does not occur here. However, for the sample with a high Cu content, a decrease in the Ni^n+^ reduction temperature is observed, which can be associated with the presence of small amounts of highly dispersed CuO that is not bound to cuprates, working according to the abovementioned mechanism.

Table 2 shows the degrees of reduction of the catalysts calculated on the basis of our experimental data. For the catalysts of the 1:1 series, such a high degree of reduction of the sample can be attributed to the partial reduction of the support surface, while for the 9:1 series, it is associated with the presence of several oxidation states of the components that are reduced sequentially (reactions 13–17).

**Figure 3:**
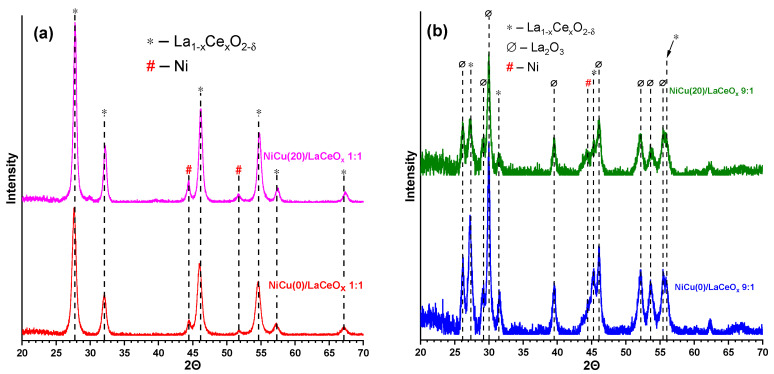
XRD patterns for Cu-Ni-containing catalysts prepared on the basis of: (**a**) LaCeO_x_ 1:1; (**b**) LaCeO_x_ 9:1, after reduction.

Figure 3 shows the XRD patterns for the reduced catalysts. The active component for the 1:1 series of catalysts is metallic Ni, and its reflections are clearly visible in the XRD patterns. The support structure does not undergo changes, revealing a LaCeO_x_ solid solution with unit cell parameters characteristic of unreduced catalysts. No reflections of Cu-containing phases are detected, which can be due to the formation of bimetallic NiCu particles with a low copper content (since there is no significant shift in the Ni(111) reflection) [38,47,49] or a highly dispersed Cu state. Table 2 shows the CSR sizes for the 1:1 catalysts.

The CSR sizes for the supports practically do not change upon reduction, while for Ni, a slight increase in the crystallite sizes relative to NiO is observed (see Table 1). Considering the higher density of Ni compared to its oxide, it can be argued that there is a strong tendency of the metal particles to sinter, which leads to a lower dispersity of the active component [52]. This is probably due to the low specific surface area of the supports and catalysts. Similar to the 1:1 series, the active component for the 9:1 samples is metallic Ni, although its reflections are rather difficult to distinguish. After the reduction, no reflections related to the phases of lanthanum nickelates or cuprates are observed; however, for all samples the formation of the La_2_O_3_ phase is observed. The phase with the active component is located on the outer surface of the LaCeO_x_ crystallites, hence the catalyst in this case is Ni/La_2_O_3_ with the partial inclusion of CeO_2_.

**Figure 4:**
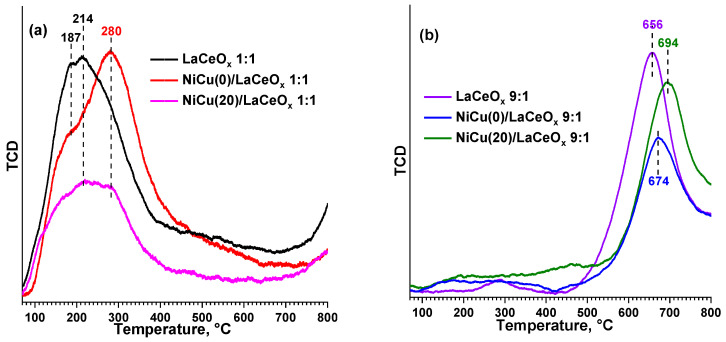
TPD-CO_2_ profiles for the supports and catalysts NiCu/LaCeO_x_: (**a**) 1:1(**b**) 9:1.

Figure 4 shows the TPD-CO_2_ data. The supports and catalysts of the 1:1 series (see Figure 4a) demonstrate a small concentration of sites with weak and medium strengths (temperature range of 200–400 °C). The absence of strong basic sites in the 1:1 samples is consistent with the retention of a crystalline structure of the CeO_2_ type in the solid solution, which lacks strong sites capable of firmly binding CO_2_ on the surface. According to the IR spectroscopy data [53], CeO_2_ is characterized by the formation of bridging, monodentate, bidentate, and polydentate carbonates; but within the specified temperature range, only bidentate (up to 200 °C) and monodentate (>200 °C) carbonates are desorbed, i.e., the species in which a dissociative adsorption with the participation of vacancies is observed [22]. An increase in the number of oxygen vacancies in the LaCeO_x_ solid solution leads to an increase in adsorption capacity, which is noticeable when compared to the pure CeO_2_ (T_max_ of desorption is 133 °C; a total of 0.0160 mmol/g CO_2_ is desorbed).

Within the 1:1 series, the following pattern is noticeable: an increase in the Cu content reduces the basic properties of the samples (Table 2, lines 1–2) due to the blocking of the adsorption sites on the catalyst surfaces. However, when no Cu is involved, the formation of stronger basic sites is observed, which is associated with the participation of Ni in the dissociative adsorption of CO_2_ [8].

Since DRM requires high temperatures (above 650 °C), at which the main properties of the 1:1 catalysts will not be exhibited, the defective structure of the LaCeO_x_, which contributes to the activation of the CO_2_ bypassing the “main” pathways and facilitating the oxidation of carbon deposition products, becomes of key importance.

Compared to the 1:1 series, the 9:1 samples (see Figure 4b) demonstrate significantly more pronounced basic properties. The temperature maxima of desorption above 650 °C indicate a high strength of the main sites and a strong CO_2_ binding; however, due to the low textural characteristics of both series, the total number of sites differs insignificantly: for the 9:1 series, the observed amount of desorbed CO_2_ on average is only 2–3 times higher than for the 1:1. For the samples with a high La_2_O_3_ content, it is generally accepted [3,5,8,17,18,26,43,44] to interpret the CO_2_ binding on the surface as a result of the formation of stable oxycarbonates (e.g., La_2_O_2_CO_3_ and La_2_CO_5_), which decompose only at high temperatures. Since DRM is implemented precisely at rather high temperatures, where strong CO_2_ binding on the surface is required, the La_2_O_3_-based catalysts can show enhanced performance, especially when using additives that increase their basic properties. Thus, according to the TPD-CO_2_ data, it can be noted that the supports and catalysts exhibit a greater strength of their main sites than those of pure La_2_O_3_ (T_max_ of desorption is 506 °C, a total of 0.3596 mmol/g CO_2_ is desorbed), but their density is much lower. This is probably due to the Ce effect, which facilitates the formation of the most reactive forms of carbonates by increasing the structure defectiveness [32], and at the same time reducing the number of adsorption sites. Within the 9:1 series, the Cu influence is ambiguous: the catalyst without copper exhibits the least basic properties, while the Cu-containing catalysts demonstrate a more pronounced strength of their basic sites. 

### 3.3. Catalytic Properties and Stability to Carbonization in DRM 

**Figure 5:**
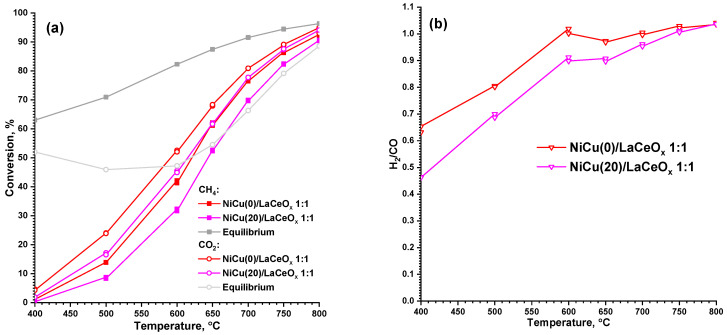
Conversions of reagents (**a**) and H_2_/CO (**b**) at 400–800 °C for the NiCu/LaCeO_x_ 1:1 catalyst series. Equilibrium conversion values of CH_4_ and CO_2_ are given according to Ref. [54].

Figure 5 shows the conversion values of both reagents in the temperature range of 400–800 °C, with the H_2_/CO ratio characterizing the activity of the 1:1 catalyst series. For all temperatures, a higher CO_2_ conversion is observed compared to CH_4_, which indicates the occurrence of side processes involving CO_2_, e.g., the reverse water gas shift reaction:

CO_2_ + H_2_ = CO + H_2_O, (18)

If one compares the conversions with the calculated equilibrium values for both reagents from the literature [54], even with a strong discrepancy between the experimental values and the theoretical ones, all trends are preserved at different temperatures within one series. The discrepancy can be explained by the existence of the system in a non-equilibrium state. At the same time, there are other equilibrium compositions [8] obtained using other parameters for thermodynamic calculations (dilution, reaction system, other carbon modifications), which are more similar to the obtained values.

Lower conversions are observed for the NiCu(20)/LaCeO_x_ 1:1 sample, which can be explained by the partial blocking of the CH_4_ activation sites on the surface of Ni and of CO_2_ at the metal–support interface, as well as the lower particle dispersion than in the samples without copper [38]. The H_2_/CO ratios that are the most similar to the desired ones are observed in the temperature range from 600 to 800 °C.

**Figure 6:**
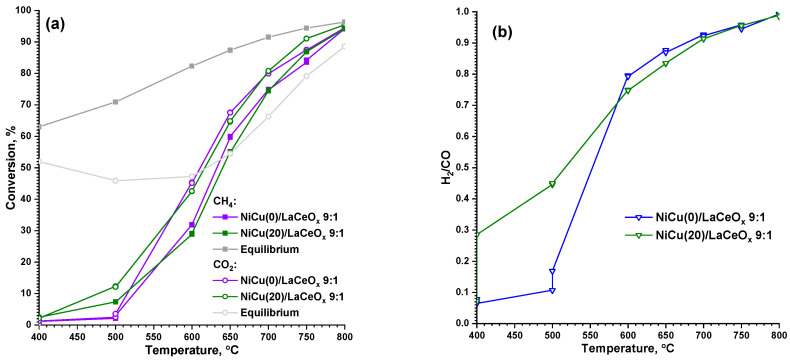
Conversions of reagents (**a**) and H_2_/CO (**b**) at 400–800 °C for the NiCu/LaCeO_x_ 9:1 catalyst series. Equilibrium conversion values for CH_4_ and CO_2_ are according to Ref. [54].

For the 9:1 catalyst series, the reagent conversions are generally comparable to those of the 1:1 series (see Figure 6a). In the region of lower temperatures (up to 650 °C), the 1:1 series demonstrates higher conversions; in the region of higher temperatures (from 650 °C and above), the conversions are almost the same. This means that the nature of the support itself does not have a significant effect on the conversion rate and that both mechanisms of CO_2_ activation are equally effective. Only the catalyst’s stability to carbon deposition is of fundamental importance.

The discrepancies among the equilibrium conversion values are of the same nature as those for the 1:1 series. For the 9:1 series, H_2_/CO ratios close to unity are achieved at higher temperatures in the region of 700–800 °C (see Figure 6b). The Cu presence does not have a noticeable effect on the reagent conversions and the H_2_/CO ratio in the 9:1 series, which allows us to conclude that Cu, in general, interacts to a lower extent with the precursor of the active component.

**Figure 7:**
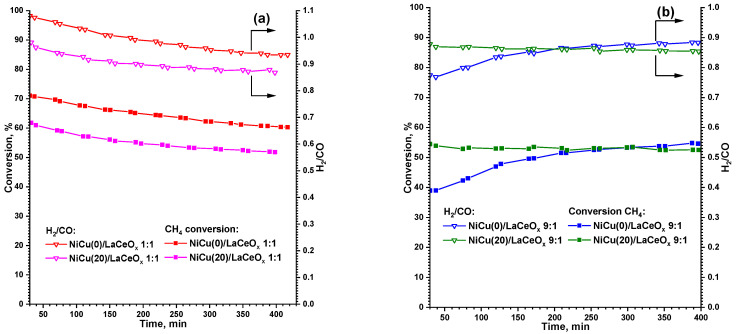
Changing of CH_4_ conversion values and H_2_/CO ratios over time at 650 °C for NiCu/LaCeO_x_ samples: (**a**) 1:1; (**b**) 9:1.

Figure 7 shows the CH_4_ conversion values (as the main source of H_2_ and carbon deposition products) and the H_2_/CO ratio depending on the time. For the 1:1 series, a gradual drop in the conversion and H_2_/CO values over time is observed, which is associated with the coking of the catalysts. For all samples, higher initial conversions and H_2_/CO ≥ 1 values are observed than at the same temperature in the gradient tests.

It can be assumed that such behavior of the samples is caused by the presence of a certain induction period, during which the predominant decomposition of CH_4_ (high methane conversions) with the formation of excess H_2_ (high H_2_/CO) is observed. In general, the samples of the 1:1 series are characterized by the same instability to coking: the drop in conversions occurs by approximately the same amount, i.e., 10–11% (see Table 3). This indicates that the Cu addition to the active component for this series of catalysts does not significantly affect the stability within the concentration range studied, but does affect the activity of the catalysts.

In the 9:1 series, the development of the NiCu(0)/LaCeOx 9:1 catalyst over time is noticeable and is accompanied by an increase in the conversions of both reagents. This is due to the presence of an induction period required for the reduction of the unreacted precursors under the influence of the reaction medium [55]:

La_2_NiO_4_ + CH_4_ = Ni + La_2_O_3_ + CO + 2H_2_, (19)

The higher stability observed after the induction period is due to the action of highly basic lanthanum oxide, which facilitates the oxidation of carbon deposition products. The sample with ~25 mol.% of Cu (exp. data, Table 1) is characterized by a slight drop in conversion over time, which is associated with a weak carbon deposition (see Table 3). Thus, the Cu addition effects the strength of the basic sites according to the TPD-CO_2_ data, and therefore the reaction rate (5) was slower in comparison to that of the NiCu(0)/LaCeO_x_ 9:1 sample. 

**Figure 8:**
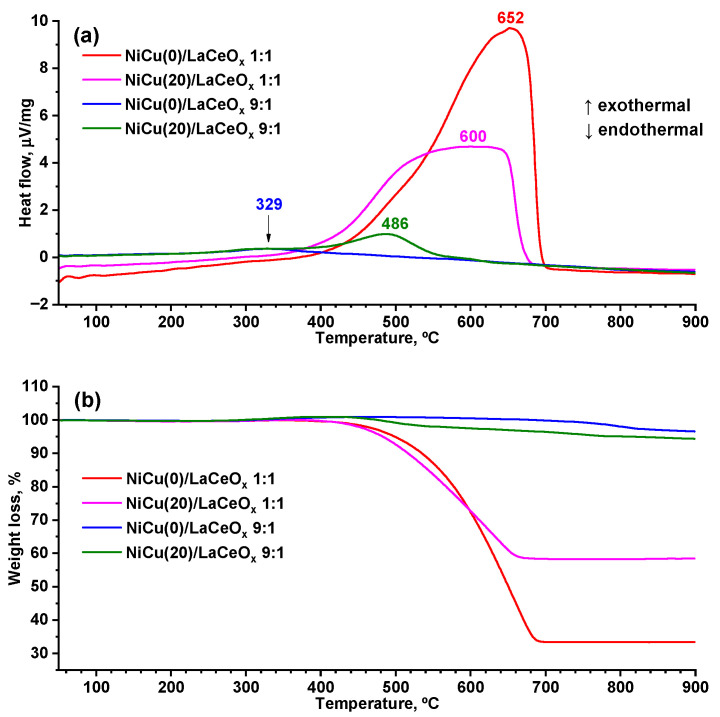
STA profiles for spent NiCu/LaCeO_x_ catalysts: (**a**) DSC curves; (**b**)TG curves.

Figure 8 shows the STA profiles for the spent catalysts.

**Figure 9:**
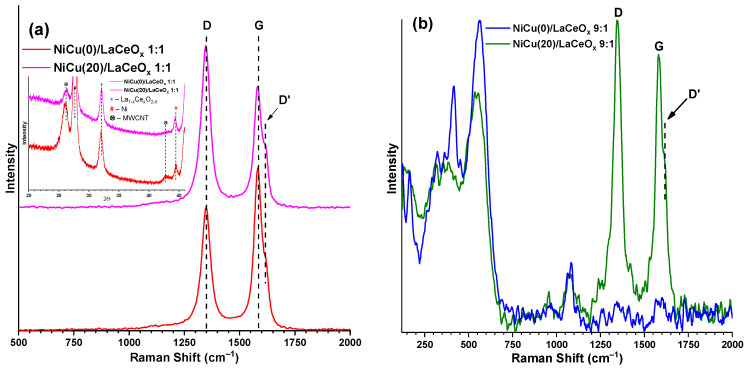
Raman spectra for spent NiCu/LaCeO_x_ catalysts: (**a**) 1:1; (**b**) 9:1.

The DSC curves for the spent catalysts of the 1:1 series feature wide carbon oxidation peaks in the temperature range of 380–700 °C. The shift in the initial temperature of carbon oxidation and the temperature maximum seen in the DSC curve can be ascribed to the differences in the crystal structure of the deposited carbon. The high temperature of burning indicates the formation of a well-organized carbon structure. This is confirmed by the Raman spectroscopy and XRD data (see Figure 9a).

The G-band (1580 cm^−1^) is characteristic of the in-plane C–C vibrations seen in such structures as graphite and graphene. The D- (1350 cm^−1^) and D’-bands (1615 cm^−1^) characterize the defectiveness of the structure; their intensity is directly proportional to the number of defects in the layered carbon structures [47]. Reflections in the diffraction patterns related to the carbon deposition products are located at 26.0° and 42.8°. The Raman spectrum and diffraction patterns are characteristic of the structures of multi-walled carbon nanotubes (MWCNT) [56,57]. This is consistent with the observed whisker-type structures for many of the carbon deposition products on Ni [8,52,58]. Table 3 shows the total amount of carbon deposition products and the characteristics of their defectiveness.

The NiCu(20)/LaCeO_x_ 1:1 sample is less susceptible to carbon deposition and forms more reactive and more defective carbon structures that can be oxidized by the active oxygen of the support, as indicated by the high I_D_/I_G_ ratio and low intensity of reflections in the carbon-containing phases and the mentioned shift in temperature maximum on the DSC curve. The sample without copper is characterized by rather strong carbon deposition with the formation of more crystallized carbon-containing products (low I_D_/I_G_ and high intensity of reflections of carbon-containing phases, relative to the Cu-containing samples). Thus, another important role of the second Cu component is that it facilitates the formation of more-defective and easily oxidized carbon-containing structures. One possible explanation for this is related to the Cu segregation on the surface of Ni particles: the resulting layer interferes with the oriented growth of nanotubes, forming disordered carbon structures.

A slightly different picture is typical for the 9:1 sample series (see Figure 9b). No carbon deposition is observed on the NiCu(0)/LaCeO_x_ 9:1 catalyst, and the one high-temperature endothermic peak can be attributed to the decomposition of lanthanum oxycarbonates (see Figure 8). This is confirmed by the absence of characteristic G- and D-bands. Minor carbon deposition is typical for NiCu(20)/LaCeO_x_ 9:1. A characteristic peak at 486 °C indicates the oxidation of carbon deposition products (see Figure 8), which is confirmed by the Raman data, indicating the formation of carbon-containing products with the structure of defective nanotubes (I_D_/I_G_ = 1.12). Vibrations in the region of 200–750 cm^−1^ are probably related to those of the support; their contribution to the overall intensity is due to the low carbon deposition on the sample, i.e., 3.8 wt.% (see Table 3). A negligible coking of the catalysts of the 9:1 series can be connected with two main reasons, including the low CH_4_ conversion rate and the presence of strong basic sites, where the oxycarbonate intermediates active in carbon oxidation are formed [59].

## 4. Conclusions

Using the wet impregnation method, the samples of bimetallic Cu-Ni catalysts supported on mixed oxides of La_2_O_3_ and CeO_2_, prepared via the citrate method with different La:Ce ratios (1:1 and 9:1) were synthesized. The La:Ce molar ratio and the introduction of the second Cu component into the active component of the catalysts were shown to have a significant effect on the support structure, the nature of the active component precursor, the acid base properties of the surface, and, as a consequence, the catalytic activity and stability to carbonization in the DRM process. The supports with the La:Ce ratio = 1:1 had a CeO_2_ fluorite crystalline structure that did not change when copper and nickel were deposited. The catalysts prepared on the basis of the supports with La:Ce = 1:1 demonstrated high catalytic activity but were prone to carbonization with the formation of multilayer nanotubes. The Cu introduction into the composition of the active component helped to increase the number of defects in the carbon structures, which improved their oxidation and reduced carbon deposition.

The samples of mono- and bimetallic catalysts with La:Ce = 9:1 were characterized by high basic properties associated with the La_2_O_3_ segregation from the solid solution and preventing the accumulation of carbon deposition products. The Cu introduction into the composition of Ni NPs contributed to the blocking of weak basic sites and insignificant carbonization compared to the samples of the 1:1 series. The lanthanum-enriched LaCeO_x_ (9:1) support prevented the accumulation of carbon deposition products on the surface of the Cu-Ni/La_2_O_3_-CeO_2_ (9:1), providing high DRM activity and a H_2_/CO ratio of 0.9.

## Figures and Tables

**Table 1:** Composition and textural properties of catalyst precursors.

Sample	ω(Ni), wt%	ω(Cu), wt%	n_La_/n_Ce_	S_BET_, m^2^/g	d_pore_, nm	Phase Composition	d(CSR), nm
LaCeO_x_ 1:1	—	—	1.0	28	8.4	La_0.54_Ce_0.46_O_2-δ_	10
NiCu(0)/LaCeO_x_ 1:1	10.4	—	1.0	20	12.6	La_0.54_Ce_0.46_O_2-δ_	12
						NiO	16
NiCu(20)/LaCeO_x_ 1:1	11.1	2.8	1.0	15	19.8	La_0.54_Ce_0.46_O_2-δ_	15
						NiO	22
LaCeO_x_ 9:1	–	–	9.8	8	19.4	La_1-x_Ce_x_O_2-δ_	22
						La_2_O_3_	20
						La_2_CO_5_	–
NiCu(0)/LaCeO_x_ 9:1	11.0	–	9.2	6	10.0	La_1-x_Ce_x_O_2-δ_	–
						La_x_NiO_y_	–
NiCu(20)/LaCeO_x_ 9:1	10.9	3.0	9.1	6	23.9	La_1-x_Ce_x_O_2-δ_	–
						La_x_NiO_y_	–

**Table 2:** Basic properties of the surfaces and features of catalyst precursor reduction in the TPR-H_2_ mode.

Sample	Total CO_2_ Consumption, mmol/g ^a^	d(CSR Ni^0^), nm		T_max_ of Reduction, °C	Experimental H_2_ Consumption, mmol/g ^b^	ω_reduction_^c^, %
NiCu(0)/LaCeO_x_ 1:1	0.091		17	195	0.050	121
				360	1.973	
				452	0.161	
NiCu(20)/LaCeO_x_ 1:1	0.043		18	220	0.446	114
				315	1.905	
				452	0.313	
NiCu(0)/LaCeO_x_ 9:1	0.138		–	385	0.756	121
				559	1.502	
NiCu(20)/LaCeO_x_ 9:1	0.144		–	348	1.407	152
				525	2.127	

^a^ Calculated based on TPR-CO_2_ data. ^b^ Calculated based on TPR-H_2_ data. ^c^ The ratio of H_2_ consumption in TPR-H_2_ to theoretical H_2_ consumption with both Ni^II^ and Cu^II^.

**Table 3:** Catalytic properties of the samples in stability tests.

Sample	X(CH_4_)/X(CO_2_), %		H_2_/CO		I_D_/I_G_	Amount of Carbon Deposition Products, mass.%
	Initial	Final, After 6 h	Initial	Final, After 6 h		
NiCu(0)/LaCeO_x_ 1:1	71.1/70.9	60.3/68.5	1.080	0.935	0.76	66.5
NiCu(20)/LaCeO_x_ 1:1	61.8/67.6	51.8/62.1	0.981	0.868	1.32	41.5
NiCu(0)/LaCeO_x_ 9:1	39.0/51.5	54.6/64.5	0.774	0.883	–	–
NiCu(20)/LaCeO_x_ 9:1	54.5/65.2	52.5/63.3	0.878	0.854	1.12	3.8

## Data Availability

The data presented in this study are available on request from the corresponding authors.

## References

[1] Lee H., Calvin K., Dasgupta D., Krinner G., Mukherji A., Thorne P., Trisos C., Romero J., Aldunce P., Barrett K. (2023). Climate Change 2023: Synthesis Report. Contribution of Working Groups I, II and III to the Sixth Assessment Report of the Intergovernmental Panel on Climate Change.

[2] Global Monitoring Laboratory, Earth System Research Laboratories Trends in Globally-Averaged CH4, N2O, and SF6 Determined from NOAA Global Monitoring Laboratory Measurements. https://gml.noaa.gov/ccgg/trends_doi.html.

[3] Pakhare D., Spivey J. (2014). A review of dry (CO_2_) reforming of methane over noble metal catalysts. Chem. Soc. Rev..

[4] Aramouni N.A.K., Touma J.G., Tarboush B.A., Zeaiter J., Ahmad M.N. (2018). Catalyst design for dry reforming of methane: Analysis review. Renew. Sust. Energ. Rev..

[5] Krylov O.V. (2000). Dry reforming of methane into syngas. Russ. J. Gen. Chem..

[6] Wang S., Lu G.Q., Millar G.J. (1996). Carbon Dioxide Reforming of Methane To Produce Synthesis Gas over Metal-Supported Catalysts: State of the Art. Energy Fuels.

[7] Fan M.S., Abdullah A.Z., Bhatia S. (2009). Catalytic Technology for Carbon Dioxide Reforming of Methane to Synthesis Gas. ChemCatChem.

[8] Papadopoulou C., Matralis H., Verykios X.E., Guczi L., Erdôhelyi A. (2012). Utilization of Biogas as a Renewable Carbon Source: Dry Reforming of Methane. Catalysis for Alternative Energy Generation.

[9] Mondal K., Sasmal S., Badgandi S., Chowdhury D.R., Nair V. (2016). Dry reforming of methane to syngas: A potential alternative process for value added chemicals—A techno-economic perspective. Environ. Sci. Pollut. Res..

[10] Yentekakis I.V., Goula G., Hatzisymeon M., Betsi-Argyropoulou I., Botzolaki G., Kousi K., Kondarides D.I., Taylor M.J., Parlett C.M.A., Osatiashtiani A. (2019). Effect of support oxygen storage capacity on the catalytic performance of Rh nanoparticles for CO_2_ reforming of methane. Appl. Catal. B.

[11] Makri M.M., Vasiliades M.A., Petallidou K.C., Efstathiou A.M. (2016). Effect of support composition on the origin and reactivity of carbon formed during dry reforming of methane over 5 wt% Ni/Ce_1−_*_x_*M*_x_*O_2−_*_δ_* (M = Zr^4+^, Pr^3+^) catalysts. Catal. Today.

[12] U.S. Geological Survey. Rare Earth Elements—Critical Resources for High Technology. https://pubs.usgs.gov/fs/2002/fs087-02/.

[13] Li P., Chen X., Li Y., Schwank J.W. (2019). A review on oxygen storage capacity of CeO_2_-based materials: Influence factors, measurement techniques, and applications in reactions related to catalytic automotive emissions control. Catal. Today.

[14] Montini T., Melchionna M., Monai M., Fornasiero P. (2016). Fundamentals and Catalytic Applications of CeO_2_-Based Materials. Chem. Rev..

[15] Liew S.Y., Jalil A.A., Tan J.S. (2022). A short review on the promotional effects of ceria-based catalyst for dry reforming methane. J. Phys. Conf. Ser..

[16] Odedairo T., Chen J., Zhu Z. (2013). Metal–support interface of a novel Ni–CeO_2_ catalyst for dry reforming of methane. Catal. Commun..

[17] Li X., Li D., Tian H., Zeng L., Zhao Z.J., Gong J. (2017). Dry reforming of methane over Ni/La_2_O_3_ nanorod catalysts with stabilized Ni nanoparticles. Appl. Catal. B.

[18] Bekheet M.F., Nezhad P.D.K., Bonmassar N., Schlicker L., Gili A., Praetz S., Gurlo A., Doran A., Gao Y., Heggen M. (2021). Steering the Methane Dry Reforming Reactivity of Ni/La_2_O_3_ Catalysts by Controlled In Situ Decomposition of Doped La_2_NiO_4_ Precursor Structures. ACS Catal..

[19] Mullins D.R. (2015). The surface chemistry of cerium oxide. Surf. Sci. Rep..

[20] Wang Y., Takahashi Y., Ohtsuka Y. (1999). Carbon Dioxide as Oxidant for the Conversion of Methane to Ethane and Ethylene Using Modified CeO_2_ Catalysts. J. Catal..

[21] Staudt T., Lykhach Y., Tsud N., Skála T., Prince K.C., Matolín V., Libuda J. (2010). Ceria reoxidation by CO_2_: A model study. J. Catal..

[22] Cheng Z., Sherman B.J., Lo C.S. (2013). Carbon dioxide activation and dissociation on ceria (110): A density functional theory study. J. Chem. Phys..

[23] Hahn K.R., Iannuzzi M., Seitsonen A.P., Hutter J. (2013). Coverage Effect of the CO_2_ Adsorption Mechanisms on CeO_2_ (111) by First Principles Analysis. J. Phys. Chem. C.

[24] de Leitenburg C., Trovarelli A., Kašpar J. (1997). A Temperature-Programmed and Transient Kinetic Study of CO_2_ Activation and Methanation over CeO_2_ Supported Noble Metals. J. Catal..

[25] Demoulin O., Navez M., Mugabo J.L., Ruiz P. (2007). The oxidizing role of CO_2_ at mild temperature on ceria-based catalysts. Appl. Catal. B.

[26] Grabchenko M., Pantaleo G., Puleo F., Kharlamova T.S., Zaikovskii V.I., Vodyankina O., Liotta L.F. (2021). Design of Ni-based catalysts supported over binary La-Ce oxides: Influence of La/Ce ratio on the catalytic performances in DRM. Catal. Today.

[27] Zhang Z., Verykios X.E., MacDonald S.M., Affrossman S. (1996). Comparative Study of Carbon Dioxide Reforming of Methane to Synthesis Gas over Ni/La_2_O_3_ and Conventional Nickel-Based Catalysts. J. Phys. Chem..

[28] Tsipouriari V.A., Verykios X.E. (2001). Kinetic study of the catalytic reforming of methane with carbon dioxide to synthesis gas over Ni/La_2_O_3_ catalyst. Catal. Today.

[29] Tsipouriari V.A., Verykios X.E. (1999). Carbon and Oxygen Reaction Pathways of CO_2_ Reforming of Methane over Ni/La_2_O_3_ and Ni/Al_2_O_3_ Catalysts Studied by Isotopic Tracing Techniques. J. Catal..

[30] Fornasiero P., Kašpar J., Graziani M. (1997). Redox Behavior of High Surface Area Rh-Loaded Ce_0.5_Zr_0.5_O_2_ Mixed Oxide. J. Catal..

[31] Zhang B., Li D., Wang X. (2010). Catalytic performance of La–Ce–O mixed oxide for combustion of methane. Catal. Today.

[32] Li X., Zhao Z.J., Zeng L., Zhao J., Tian H., Chen S., Li K., Sangab S., Gong J. (2018). On the role of Ce in CO_2_ adsorption and activation over lanthanum species. Chem. Sci..

[33] Kim S.M., Abdala P.M., Margossian T., Hosseini D., Foppa L., Armutlulu A., van Beek W., Comas-Vives A., Copéret C., Müller C. (2017). Cooperativity and Dynamics Increase the Performance of NiFe Dry Reforming Catalysts. J. Am. Chem. Soc..

[34] Wu Z., Yang B., Miao S., Liu W., Xie J., Lee S., Pellin M.J., Xiao D., Su D., Ma D. (2019). Lattice Strained Ni-Co alloy as a High-Performance Catalyst for Catalytic Dry Reforming of Methane. ACS Catal..

[35] Passos A.P., Pulcinelli S.H., Santilli C.V., Briois V. (2019). Operando monitoring of metal sites and coke evolution during non-oxidative and oxidative ethanol steam reforming over Ni and NiCu ex-hydrotalcite catalysts. Catal. Today.

[36] Lee J.H., Lee E.G., Joo O.S., Jung K.D. (2004). Stabilization of Ni/Al_2_O_3_ catalyst by Cu addition for CO_2_ reforming of methane. Appl. Catal. A.

[37] Halliche D., Bouarab R., Cherifi O., Bettahar M.M. (1996). Carbon dioxide reforming of methane on modified Ni/α-Al_2_O_3_ catalysts. Catal. Today.

[38] Chatla A., Ghouri M.M., El Hassan O.W., Mohamed N., Prakash A.V., Elbashir N.O. (2020). An experimental and first principles DFT investigation on the effect of Cu addition to Ni/Al_2_O_3_ catalyst for the dry reforming of methane. Appl. Catal. A.

[39] Zambaldi P., Haug L., Penner S., Klötzer B. (2022). Dry Reforming of Methane on NiCu and NiPd Model Systems: Optimization of Carbon Chemistry. Catalysts.

[40] Danks A.E., Hallb S.R., Schnepp Z. (2016). The evolution of ‘sol–gel’ chemistry as a technique for materials synthesis. Mater. Horiz..

[41] Livage J., Henry M., Sanchez C. (1998). Sol-gel chemistry of transition metal oxides. Prog. Solid State Chem..

[42] Bularzik J., Davies P.K., Navrotsky A. (1986). Thermodynamics of Solid-Solution Formation in NiO-CuO. J. Am. Ceram. Soc..

[43] Gallego G.S., Mondragón F., Barrault J., Tatibouët J.M., Batiot-Dupeyrat C. (2006). CO_2_ reforming of CH_4_ over La–Ni based perovskite precursors. Appl. Catal. A.

[44] Grabchenko M., Pantaleo G., Puleo F., Vodyankina O., Liotta L.F. (2021). Ni/La_2_O_3_ catalysts for dry reforming of methane: Effect of La_2_O_3_ synthesis conditions on the structural properties and catalytic performances. Int. J. Hydrogen Energy.

[45] Rojas M.L., Fierro J.L.G., Tejuca L.G., Bell A.T. (1990). Preparation and characterization of LaMn_1−_*_x_*Cu*_x_*O_3+λ_ perovskite oxides. J. Catal..

[46] Touahra F., Rabahi A., Chebout R., Boudjemaa A., Lerari D., Sehailia M., Halliche D., Bachari K. (2016). Enhanced catalytic behaviour of surface dispersed nickel on LaCuO_3_ perovskite in the production of syngas: An expedient approach to carbon resistance during CO_2_ reforming of methane. Int. J. Hydrogen Energy.

[47] Ashok J., Reddy P.S., Raju G., Subrahmanyam M., Venugopal A. (2009). Catalytic Decomposition of Methane to Hydrogen and Carbon Nanofibers over Ni−Cu−SiO_2_ Catalysts. Energy Fuels.

[48] Xie Y., Chen J., Wu X., Wen J., Zhao R., Li Z., Tian G., Zhang Q., Ning P., Hao J. (2022). Frustrated Lewis Pairs Boosting Low-Temperature CO_2_ Methanation Performance over Ni/CeO_2_ Nanocatalysts. ACS Catal..

[49] Lázaro M.J., Echegoyen Y., Suelves I., Palacios J.M., Moliner R. (2007). Decomposition of methane over Ni-SiO_2_ and Ni-Cu-SiO_2_ catalysts: Effect of catalyst preparation method. Appl. Catal. A.

[50] da Silva A.A.A., da Costa L.O.O., Mattos L.V., Noronha F.B. (2013). The study of the performance of Ni-based catalysts obtained from LaNiO_3_ perovskite-type oxides synthesized by the combustion method for the production of hydrogen by reforming of ethanol. Catal. Today.

[51] Falcón H., Martinez-Lope M.J., Alonso J.A., Fierro J.L.G. (2000). Defect LaCuO_3−_*_δ_* (*δ* = 0.05−0.45) perovskites: Bulk and surface structures and their relevance in CO oxidation. Appl. Catal. B.

[52] Li M., van Veen A.C. (2018). Tuning the catalytic performance of Ni-catalysed dry reforming of methane and carbon deposition via Ni-CeO_2-_*_x_* interaction. Appl. Catal. B.

[53] Binet C., Daturi M., Lavalley J.C. (1999). IR study of polycrystalline ceria properties in oxidised and reduced states. Catal. Today.

[54] Nikoo M.K., Amin N.A.S. (2011). Thermodynamic analysis of carbon dioxide reforming of methane in view of solid carbon formation. Fuel Process. Technol..

[55] Batiot-Dupeyrat C., Valderrama G., Meneses A., Martinez F., Barrault J., Tatibouët J.M. (2003). Pulse study of CO_2_ reforming of methane over LaNiO_3_. Appl. Catal. A.

[56] Chen Y., Tao J., Ezzeddine A., Mahfouz R., Al-Shahrani A., Alabedi G., Khashab N.M. (2015). Superior Performance Nanocomposites from Uniformly Dispersed Octadecylamine Functionalized Multi-Walled Carbon Nanotubes. C.

[57] He X., Xu X., Boa G., Yan Y. (2020). Studies on the effects of different multiwalled carbon nanotube functionalization techniques on the properties of bio-based hybrid non-isocyanate polyurethane. RSC Adv..

[58] Al-Sabban B.E.A. (2016). Development of Coke-Tolerant Transition Metal Catalysts for Dry Reforming of Methane. Ph.D. Thesis.

[59] Zhou R., Mohamedali M., Ren Y., Lu Q., Mahinpey N. (2023). La-Ce binary oxide catalysts for low-temperature dry reforming. Int. J. Hydrogen Energy.

